# Enzyme Mimetic Activity of ZnO-Pd Nanosheets Synthesized via a Green Route

**DOI:** 10.3390/molecules25112585

**Published:** 2020-06-02

**Authors:** Ravi Mani Tripathi, Dohee Ahn, Yeong Mok Kim, Sang J. Chung

**Affiliations:** 1 School of Pharmacy, Sungkyunkwan University, 2066 Seoburo, Jangan-gu, Suwon, Gyeonggido 16419, Korea; rmtripathi02@gmail.com (R.M.T.); ehgml94@naver.com (D.A.); rusynante@naver.com (Y.M.K.); 2 Amity Institute of Nanotechnology, Amity University Uttar Pradesh, Sector 125, Noida 201303, India

**Keywords:** green synthesis, *Erigeron annuus* leaf extract, ZnO-Pd nanosheets, peroxidase mimetic activity, nanozyme

## Abstract

Recent developments in the area of nanotechnology have focused on the development of nanomaterials with catalytic activities. The enzyme mimics, nanozymes, work efficiently in extreme pH and temperature conditions, and exhibit resistance to protease digestion, in contrast to enzymes. We developed an environment-friendly, cost-effective, and facile biological method for the synthesis of ZnO-Pd nanosheets. This is the first biosynthesis of ZnO-Pd nanosheets. The synthesized nanosheets were characterized by UV–visible spectroscopy, X-ray diffraction (XRD), scanning electron microscopy, transmission electron microscopy, and energy-dispersive X-ray. The d-spacing (inter-atomic spacing) of the palladium nanoparticles in the ZnO sheets was found to be 0.22 nm, which corresponds to the (111) plane. The XRD pattern revealed that the 2θ values of 21.8°, 33.3°, 47.7°, and 56.2° corresponded with the crystal planes of (100), (002), (112), and (201), respectively. The nanosheets were validated to possess peroxidase mimetic activity, which oxidized the 3,3′,5,5′-tetramethylbenzidine (TMB) substrate in the presence of H_2_O_2_. After 20 min of incubation time, the colorless TMB substrate oxidized into a dark-blue-colored one and a strong peak was observed at 650 nm. The initial velocities of Pd-ZnO-catalyzed TMB oxidation by H_2_O_2_ were analyzed by Michaelis–Menten and Lineweaver–Burk plots, resulting in 64 × 10^−6^ M, 8.72 × 10^−9^ Msec^−1^, and 8.72 × 10^−4^ sec^−1^ of *K*_M_, *V*_max_, and *k*_cat_, respectively.

## 1. Introduction

Nanomaterials have drawn attention owing to their unique optical, electronic, magnetic, and catalytic properties, and are widely used in various fields, including as catalysts [1,2,3,4], photocatalysts [5], antibacterials [6,7,8], colorimetric sensors [9], and drug delivery systems [10,11]. In general, however, chemical methods applied for the synthesis of nanomaterials have created environmental pollution, as hazardous chemicals are required, whereas the synthesis of nanomaterials using biological or green routes may lead to the development of clean, non-toxic, and environmentally friendly “green chemistry” procedures by involving plant extracts and organism biomasses ranging from bacteria to fungi. Plant extracts have gained substantial consideration compared to microorganism biomasses of fungi and bacteria because there are no requirements for specific conditions, media, or culture maintenance. Several nanomaterials have already been biosynthesized, such as nanoparticles [3,6,12,13], carbon dots [14], nanoflowers [2,5], alloys [1], and nanofibers [15].

Enzymes are good catalysts in many biological processes [16], but require specific physiological conditions to perform their optimal catalytic activity. The peroxidase enzyme catalyzes the oxidation of the substrate in the presence of hydrogen peroxide (H_2_O_2_), acting as electron acceptor. Horseradish peroxidase (HRP) has outstanding properties, which makes it suitable for various applications. A wide range of substrates can be oxidized by HRP, such as phenols, indoles, aromatic amines, and sulfonates. Chemical cross-linking, freeze drying, and prolonged storage at 4 °C do not have any effect on the stability and function of HRP [17]. HRP can polymerize the aromatic substrates, which is its most prominent application in the removal of aromatic pollutants from water [18]. HRP is the most widely used enzyme in various biochemical applications. Several methods have been developed for the investigation of the enzyme activity of peroxidase-labelled immunoreagents, such as chemiluminescence, colorimetry, and fluorimetry [19]. HRP is also used in medical diagnostic, biosensing, bioremediation, and biotechnological applications [20,21]. The detection is based on the redox reaction mechanism of HRP, which regulates the conversion of the colorless 3,3′,5,5′-tetramethylbenzidine (TMB) substrate into an oxidized blue-green-colored, single electron loss oxidation state product. The drawbacks of enzymes are the high costs of synthesis, isolation, and purification, and their limited stability in harsh environments. Therefore, a recent development in the area of nanotechnology focused on the development of nanomaterials that exhibit enzyme-like activities. Over the past few years, researchers have developed artificial alternatives to enzymes with high stability. The enzyme-mimicking nanozymes work efficiently as catalysts in extreme conditions of pH and temperature, and also demonstrate resistance to protease digestion. Among these examples, enzyme-mimicking nanomaterials have gained more importance in the case of horseradish peroxidase due to their high surface-to-volume ratios; the presence of large surface activation centers; and their easily controllable size, shape, and surface charge. Several researchers have developed nanomaterials based on peroxidase-mimicking nanozymes, such as carbon nanotubes [22], carbon dots [23], graphene oxide [24], sheet-like and spherical FeS nanostructures [25], bimetallic alloy nanostructures [26], gold nanoparticles [27], and hemin–graphene oxide (GO) hybrid nanosheets [28]. These enzyme mimics are more stable than their natural counterparts, with comparatively simple and cost-effective preparation and storage.

Earlier, we proposed an unprecedented method for the synthesis of ZnO nanoflowers, which exhibit good photocatalytic activity [5]. With a slight modification, we now present a biological method for the synthesis of ZnO-Pd nanosheets. For this synthesis, *Erigeron annuus* (*E.* *annuus*) leaf extract was used. The leaf extract of *E. annuus* contains γ-pyranone derivatives, flavonoids, and phenolic acids [29]. These biomolecules actively participate in the synthesis of nanomaterials [12,30]. The synthesized ZnO-Pd nanosheets were characterized by ultraviolet–visible (UV–vis) spectroscopy, scanning electron microscopy (SEM), transmission electron microscopy (TEM), energy-dispersive X-ray spectroscopy (EDX), and X-ray diffraction (XRD). We observed that the palladium nanoparticles played a significant role in the oxidation of the peroxidase substrate TMB (colorless) to the oxidized TMB (oxTMB, blue). Generally, pure nanoparticles are agglomerated and aggregated after the use of ligand during the application. Therefore, the use of a matrix to fix the nanoparticles is a promising approach to avoid the agglomeration and aggregation of nanoparticles. Thus, ZnO nanosheets were used as a matrix to fix the PdNPs and prevent agglomeration and aggregation of PdNPs during the catalytic oxidation of the peroxidase substrate TMB. The FTIR result showed that the presence of biomolecules from the leaf extract on the surface of nanosheets prevented agglomeration and aggregation. The influence of surface properties on catalytic activity is one of the major concerns for active sites. Liu et. al. (2019) studied the sulfide ion (S^2−^)-induced inhibition of the peroxidase-like activity of gold nanoparticles (AuNPs) to explore active sites [31]. The sulfide ion (S^2−^) had interacted with Au(I) and formed Au_2_S, which suggested that Au(I) could be the active site of AuNPs for the peroxidase-like activity. They observed by X-ray photoelectron spectroscopy that the addition of S^2−^ decreased the Au(I) on the surface of AuNPs. Therefore, the presence of Au(I) on the surface of AuNPs is the requisite that provides the active site for peroxidase-like activity. In this study, we evaluate the peroxide-like activity of ZnO nanosheets and ZnO-Pd nanosheets. The results show that the ZnO-Pd nanosheets have excellent peroxidase-like activity, because the Pd provides an active site for this activity. Michaelis–Menten and Lineweaver–Burk plots were used to determine the initial velocities of Pd-ZnO-catalyzed TMB oxidation by H_2_O_2_, showing values of 64 × 10^−6^ M, 8.72 × 10^−9^ Msec^−1^, and 8.72 × 10^−4^ sec^−1^ for *K*_M_, *V*_max_, and *k*_cat_, respectively. The substrate specificity (*k*_cat_/*K*_M_) was found to be 13.6 M^−1^sec^−1^. The synthesized nanosheets show excellent peroxidase activity in vitro. This is the first report on the biosynthesis of ZnO-Pd nanosheets with high peroxidase activity by a green synthesis method.

## 2. Results and Discussion

### 2.1. UV–Vis Spectroscopy Analysis

UV–vis spectroscopy is a widely used technique to analyze the optical properties of nanostructured materials. The UV–vis absorption of as-synthesized ZnO and ZnO-Pd nanosheets was recorded at wavelengths of 250–700 nm (Figure S1a,c). At the end of the biosynthesis of ZnO nanosheets, a white-colored precipitate was produced, indicating the formation of ZnO (Figure S1b). The UV–vis spectrum of ZnO nanosheets exhibited absorption at approximately 375 nm. However, the absorption spectrum of as-synthesized ZnO-Pd nanosheets exhibited no peak, because the peak was removed by the formation of zero valent palladium. The biosynthesis of ZnO-Pd nanosheets resulted in a mixed-colored precipitate (light yellow-brown), indicating the formation of ZnO-Pd nanosheets (Figure S1d). In a previously reported study, the synthesis of the ZnO nanostructure was accomplished in 48 h using bacterial biomass [5], whereas the present method needed less than 160 min for the synthesis using leaf extracts. Therefore, the present method is faster than the previous one that used bacterial biomass.

### 2.2. XRD Analysis

**Figure 1:**
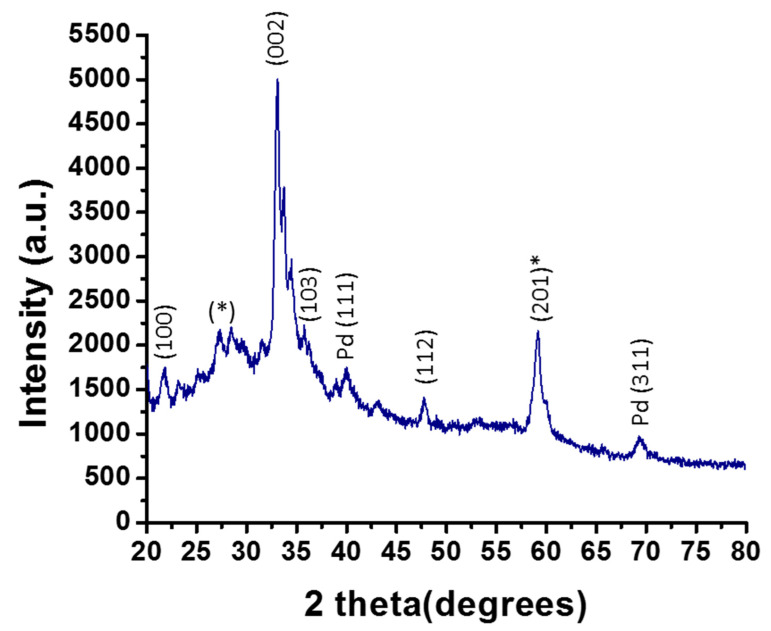
XRD pattern of ZnO-Pd nanosheet biosynthesis (٭-the reflections present in the ZnO-Pd nanosheets, which may have been caused by the residual moieties of the leaf extract).

**Figure 2:**
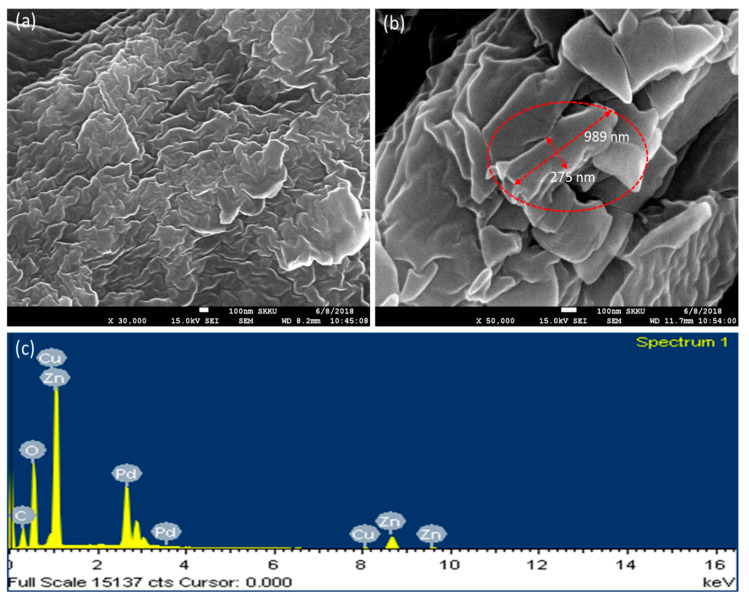
Field emission scanning electron microscope (FESEM) images of biosynthesized ZnO-Pd nanosheets. (**a**) Overall view of the sample. (**b**) Micrograph showing the nanosheets. (**c**) Energy-dispersive X-ray (EDX) spectrum of biosynthesized ZnO-Pd nanosheets.

XRD patterns of the ZnO-Pd nanosheets were determined to understand their crystal structure and purity (Figure 1). The diffraction peaks were in good agreement with the hexagonal phase of ZnO (The Joint Committee on Powder Diffraction Standards data card 89-0510) [32]. The 2θ values were 21.8°, 33.3°, 47.7°, and 56.2° (the peak shifted at 59.1° due to the composite of palladium), corresponding to the crystal planes of (100), (002), (112), and (201), respectively (Figure 2). The ZnO peaks in the XRD pattern appeared with palladium, with an extra low-intensity peak at an approximate 2θ value of 40.05°, corresponding to the crystal plane (111) of Pd (JCPDS no. 87-0641), which confirmed the presence of palladium in the nanosheets (Figure 1). Another peak was observed at an approximate 2θ value of 68.5°, corresponding to the crystal plane (311) of palladium. Two peaks at 2θ values of 28.47° and 29.49° were also found in addition to the reflections present in the ZnO-Pd nanosheets, which may have been caused by the residual moieties of the leaf extract.

### 2.3. Field Emission Scanning Electron Microscope Analysis

**Figure 3:**
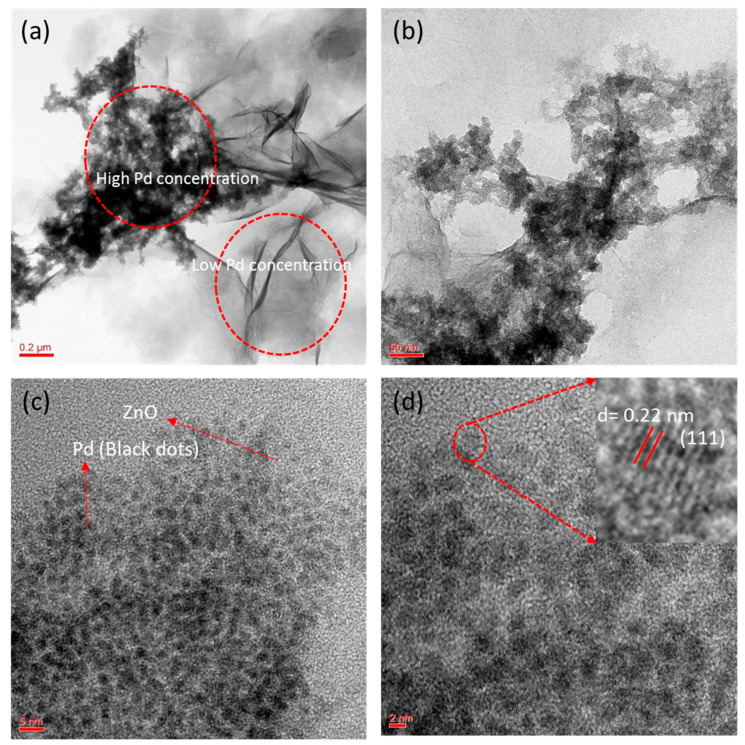
Transmission electron microscopy (TEM) images of biosynthesized ZnO-Pd nanosheets. (**a**) Overall view of sample. (**b**) Magnified view of nanosheets. (**c**) High-resolution (HR) TEM micrographs. (**d**) HR-TEM micrograph showing the crystalline nature (the inset shows the d-spacing of the palladium nanoparticles).

A field emission scanning electron microscope (FESEM) was used to analyze the morphology and size of the ZnO-Pd nanosheets. Freeze-dried ZnO-Pd nanosheets were used to prepare a sample for FESEM analysis. Figure 2a,b depict the nanosheet-like structures of ZnO-Pd. Figure 3a depicts a large view of the sample taken at 30,000× magnification, which reveals the uniformity in the nanosheets. Furthermore, the sample was scanned at 50,000× magnification, and a large length of approximately 989 nm and width of approximately 275 nm (red circle) were observed (Figure 2b). Therefore, the SEM micrographs clearly revealed that the synthesized ZnO-Pd nanosheets have a sheet-like morphology.

### 2.4. TEM Analysis

We analyzed the sample from FESEM and found that sheet-like ZnO-Pd nanosheets had been synthesized, but no information was available regarding the palladium position in the nanosheets due to the low magnification of FESEM. Therefore, the sample was further characterized to confirm the morphology and palladium position in the nanosheets. A TEM sample of as-synthesized ZnO-Pd nanosheets was prepared on a carbon-coated grid by drop coating method. The TEM micrographs show that the synthesized ZnO-Pd has a sheet-like structure, with the palladium particles incorporated in it (Figure 3a–d), which is quite consistent with the FESEM results. Figure 3a depicts the nanosheet structures with low and high palladium concentrations. Another micrograph was taken at high magnification at the 50 nm scale, showing that the palladium particles are agglomerated (Figure 3b). High-resolution TEM micrographs show that palladium particles in the ZnO nanosheets are well dispersed and no agglomeration can be observed (Figure 3c,d). Figure 3c depicts monodispersed palladium particles present in the nanosheets, with a size of approximately 2.5 nm. The high-resolution TEM micrographs also show that the palladium nanoparticles exhibit the lattice fringe characteristics of crystalline materials (Figure 3d). The d-spacing (inter-atomic spacing) of the palladium nanoparticles is found to be 0.22 nm, which corresponds to the (111) plane (inset Figure 3d). Therefore, the present biological method is supported by the noble quality synthesis of ZnO-Pd nanosheets. This is the first biosynthesis report of the development of a novel, eco-friendly, and cost-effective synthesis method for ZnO-Pd nanosheets. However, a chemical method has been developed for synthesis of ZnO nanosheets, which requires methyl alcohol and *p*-xylene and refluxing at 80 °C for 12 h [33]. However, the present synthesis method requires leaf extract, NaOH, and no reflux. Furthermore, the present synthesis method needs a temperature of 60 °C and 160 min to complete the synthesis. The synthesized ZnO-Pd nanosheets were freeze-dried to make powder and stored at room temperature for future application. The powder form of ZnO-Pd nanosheets was characterized by TEM after 7 months and no agglomeration was observed, showing that the synthesized nanosheets have high stability. Therefore, the present synthesis method for nanosheets is cost-effective and environmentally friendly.

### 2.5. EDX Analysis

The elemental composition of ZnO-Pd nanosheets was analyzed by EDX. The EDX spectrum was obtained during the FESEM analysis of the nanosheets, as the EDX device is an attachment of the SEM or TEM instrument. Figure 2c depicts the EDX elemental profile of ZnO-Pd nanosheets, which displays strong elemental signals of Zn, O, and Pd, demonstrating their presence in the synthesized ZnO-Pd nanosheets, as no other elemental signals were noticed in the EDX, excluding those of Cu and C. The occurrence of the Cu and C signals was caused by the sample grid. Therefore, the biosynthesized ZnO-Pd nanosheets contained pure elemental Zn, O, and Pd.

**Figure 4:**
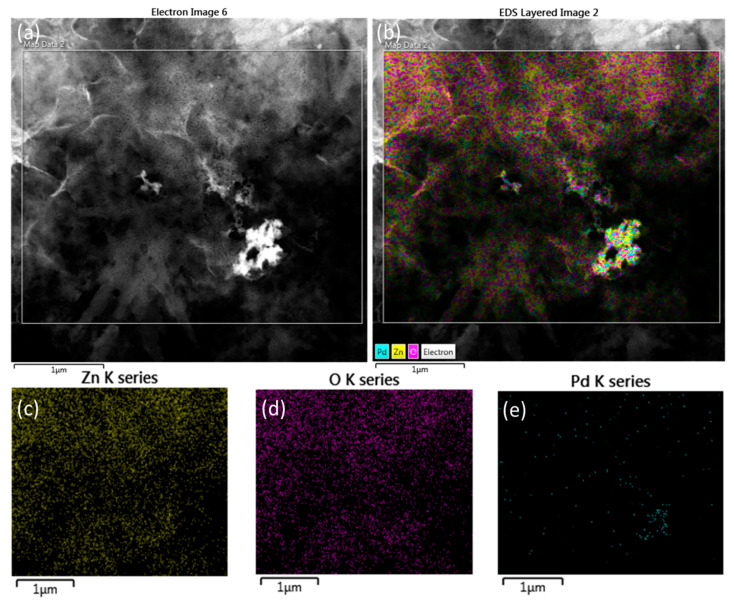
EDX mapping of biosynthesized ZnO-Pd nanosheets. (**a**) Area selected in the TEM image for the elemental mapping. (**b**) Distribution of Zn, O, and Pd elements. (**c**) Zn–K map (yellow). (**d)** O-K map (magenta). (**e**) Pd-K map (blue).

The EDX spectroscopy of the hybrid ZnO-Pd nanosheets was also performed with the TEM in the elemental mapping (EDS mapping) mode (Figure 4a–e). Figure 4a depicts the selected area in the TEM micrograph for EDS mapping. A homogenous distribution of Zn, O, and Pd elements was observed in the EDS mapping of the ZnO-Pd nanosheets (Figure 4b). A uniform distribution of Pd was seen in the nanosheets. The observed Zn and O element distribution was broader than that of elemental Pd, which indicates that Pd is compactly encapsulated in the ZnO lattice (Figure 4c–e). The elemental Zn, O, and Pd are highly dispersed in the nanosheets, confirming that no other impurities exist in the sheets. Therefore, the EDX profile and mapping confirmed the hybrid nature of the ZnO-Pd nanosheets.

### 2.6. FTIR Analysis

The biosynthesized nanosheets were analyzed by FTIR to assess the contribution of biological molecules. Figure S2 show the FTIR spectrum of biosynthesized nanosheets in the scanning range of 650 to 4000 cm^−1^. A broad and strong peak was observed at 3448.1 cm^−1^, corresponding to the -OH stretching vibrations of the OH units and water [34]. A medium peak was observed at 2973.7 cm^−1^, which corresponded to C-H stretching vibrations of alkane. The peak at 2868.59 cm^−1^ indicates the bond for C-H stretching (2830–2695 cm^–1^), which corresponds to aldehyde. The absorption peak at 2300–2400 cm^−1^ was attributed to the stretching of C=N bond [35]. The peak found at 1648.84 cm^−1^ shows the bond for (N–H) bending, which corresponds to primary amines. The bending of the C–H aldehyde bonds was observed at 1396.21 cm^−1^. Lastly, a medium peak at 1056.8 cm^−1^ corresponded to the flavanones adsorbed on the surface of the nanosheets [36]. The FTIR spectrum showed that the biological molecules in the leaf extract were formed during the synthesis of the nanosheets.

### 2.7. Peroxidase Mimetic Activity

Several clinical diagnoses have used the chromogenic substrate TMB. The peroxidase enzyme oxidizes TMB in the presence of H_2_O_2_ and gives color. However, enzyme production and purification are extremely expensive and time consuming. Moreover, enzymes do not work in harsh pH and temperature conditions. Therefore, we developed ZnO-Pd nanosheets for their peroxidase mimetic activity. In a typical reaction, 0.12 mg/mL ZnO-Pd nanosheets are used to oxidize 0.525 mM TMB in the presence of 20 mM H_2_O_2_. The reaction was performed in acetate buffer with a pH of 4. After 20 min of incubation at room temperature, the nanosheets produced a blue color. The UV–vis spectra for both normal and oxidized TMB were measured and a strong peak at 650 nm was observed, clearly indicating the characteristic of oxidized TMB (Figure S3).

### 2.8. Comparison of Peroxidase Mimetic Activity of ZnO and ZnO-Pd Nanosheets

**Figure 5:**
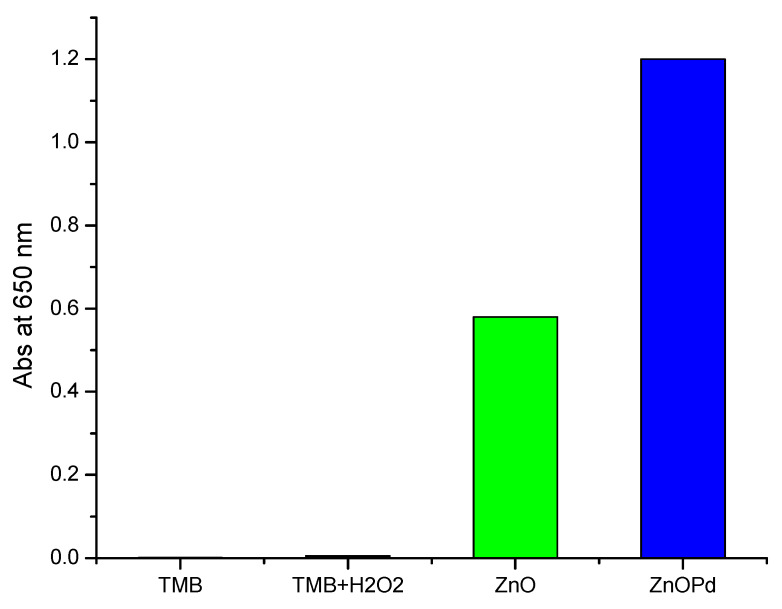
Catalytic comparison of 3,3′,5,5′-tetramethylbenzidine (TMB) oxidized by ZnO and ZnO-Pd nanosheets by measuring the absorbance at 650 nm.

Previous studies have reported that nanocomposites or alloy nanomaterials exhibit high catalytic activity in comparison with pure materials [1,37]. We analyzed the peroxidase mimetic activity of the biosynthesized ZnO and ZnO-Pd nanosheets. Both types of synthesized nanosheets were taken in equal concentrations for the catalytic comparison. TMB treated with ZnO-Pd nanosheets showed exactly double the absorption at 650 nm in comparison with the ZnO nanosheet treatment (Figure 5). Therefore, due to the oxidizing power of ZnO-Pd nanosheets in the presence of H_2_O_2_, they demonstrated higher catalytic activity in comparison with simple ZnO nanosheets.

### 2.9. Influence of Incubation Time on Peroxidase Mimetic Activity

**Figure 6:**
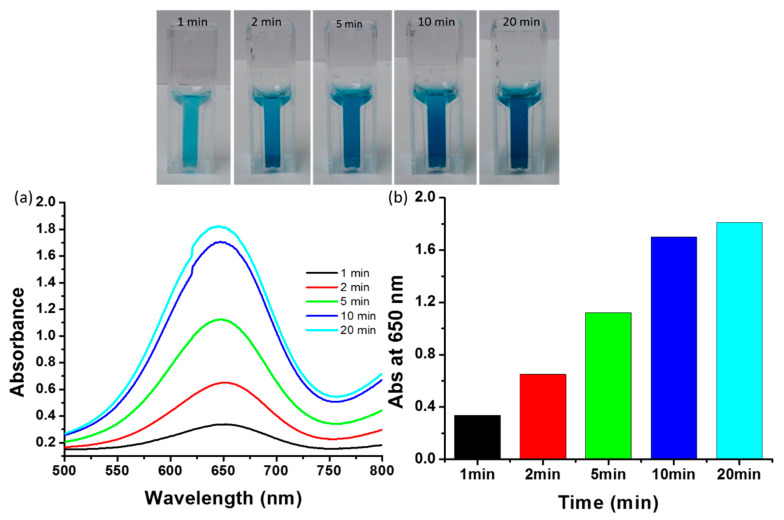
Effect of incubation time. (**a**) Absorbance spectra at various incubation times and (inset) cuvettes demonstrating the effect of incubation time on the color development. (**b**) Absorbance at 650 nm with the function of time.

Incubation time plays a significant role in the oxidation of TMB when using biosynthesized ZnO-Pd nanosheets. A typical reaction mixture contained 0.525 mM TMB, 20 mM H_2_O_2_, and 0.12 mg/mL ZnO-Pd nanosheets. The acetate buffer was used as a medium to maintain the pH of the reaction mixture. The color of the reaction mixture intensified with increasing incubation time. The cuvettes showed that the dark blue color developed after 10 min and 20 min of incubation time, respectively, for TMB and oxidized TMB (Figure 6 inset). We observed that the cuvettes at 10 min and 20 min showed a more or less similar color intensity. The UV–vis spectra were also recorded for the quantitative analysis of the oxidation of TMB (Figure 6a). Figure 6b reveals that the absorption increased at 650 nm as a function of incubation time. Rapid growth in absorption was observed until 10 min of incubation, with little difference thereafter up to 20 min. Therefore, we conclude that the complete oxidation of 0.525 mM TMB was accomplished in 20 min of incubation time. The incubation time of the assay depended on the type of catalysts used. The enzyme ficin and a zinc(II)-2-methylimidazole metal organic framework showed enhanced peroxidase activity, but the procedure needed 180 min of incubation time [38]. Chen et. al. (2017) reported that molybdenum disulfide (MoS_2_) nanosheets show peroxidase-like activity and require 30 min optimum incubation time [39], whereas as-synthesized ZnO-Pd requires only 20 min incubation time.

### 2.10. Determination of Catalytic Activity for TMB Oxidation

The amounts of Pd and Zn in Pd/ZnO nanosheets were determined to be 0.45% and 11.6%, respectively, by ICP-MS analysis; while the other components were estimated based on the spectral data to be the organic materials, inorganic metal salts, and water originating from the plant extract (Table S1). Based on the analytical data, the amount of Pd used in the TMB oxidation was calculated.

**Figure 7:**
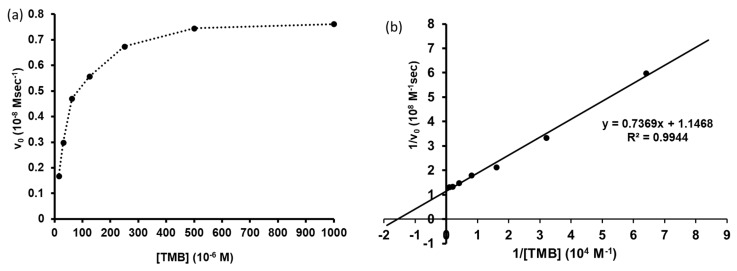
Steady-state kinetics assay of ZnO-Pd. The initial velocity of TMB oxidation was monitored for 3 min after adding ZnO-Pd (10 μM Pd) into TMB (1000, 500, 250, 125, 62.5, 31.25, 15.625 μM) and H_2_O_2_ in pH 5.0, 0.1 M acetate buffer supplemented with 20% DMSO. The *K*_M_ (6.4 x 10^−5^ M), *V_max_* (8.72 × 10^−9^ Msec^−1^), and *k*_cat_ (8.72 × 10^−4^ sec^−1^) were determined by analysis of Michaelis–Menten (**a**) and Lineweaver–Burk plots (**b**).

The oxidoreductase activity of Pd-ZnO nanosheets was determined by measuring the change in absorption at 650 nm, resulting from TMB oxidation by H_2_O_2_ [40]. The slope was converted to initial velocity (M/sec) using the extinction coefficient (*ε*_650_ = 3.9 × 10^4^ M^−1^cm^−1^) of oxidized TMB [41]. The plot of the initial velocity (*v*_0_) over reactant concentrations ([S]) showed a saturation curve, suggesting a pseudo-first-order reaction. The initial velocities of Pd-ZnO-catalyzed TMB oxidation by H_2_O_2_ were analyzed by Michaelis–Menten and Lineweaver–Burk plots, resulting in 64 × 10^−6^ M, 8.72 × 10^−9^ Msec^−1^ and 8.72 × 10^−4^ sec^−1^ for *K*_M_, *V*_max_, and *k*_cat_, respectively (Figure 7). The substrate specificity (*k*_cat_/*K*_M_) was determined to be 13.6 M^−1^sec^−1^. As we cannot currently determine the amount of surface-exposed Pd catalyst, the exact *k*_cat_ and *k*_cat_/K_M_ values were not available but were estimated based on the total amount of Pd used in the reactions. These results suggest that Pd-ZnO nanosheets would work as nanozymes [42]. Considering that the amounts of Pd and ZnO were only 0.45% and 11.6%, the residual materials in the nanosheets would be organic materials, including proteins, carbohydrates, organic chemicals, and inorganic salts originating from plant extracts. It is suggested, therefore, that the hydrophobic materials, such as proteins, carbohydrates, and organic molecules, surround Pd and ZnO and interact with TMB, so that the enzyme active site catches its substrate.

## 3. Materials and Methods 

### 3.1. Materials

Zinc acetate dihydrate and palladium (II) chloride were bought from Sigma–Aldrich (St. Louis, MO, USA) and used as precursors for the synthesis. The chromogenic substrate 3,3′,5,5′-tetramethylbenzidine (TMB) was purchased from Sigma–Aldrich for analysis of the peroxidase mimetic activity of the nanosheets. Hydrogen peroxide (H_2_O_2_) was purchased from Samchun Chemical Co., Ltd. (Seoul, South Korea). All other chemicals were analytical-grade and used as received, without purification. Deionized (DI) water was used in all the experiments related to the biosynthesis of the nanoparticles and for analyzing the peroxidase mimetic activity of the nanosheets.

### 3.2. E. annuus Leaf Extract Preparation

Fresh leaves of *E. annuus* were collected from the campus of Sungkyunkwan University, Gyeonggi-do, Korea. The leaves were thoroughly washed first with tap water and subsequently with deionized (DI) water. The washed leaves were dried at room temperature to remove the adsorbed water, then cut into small pieces, and 8.5 g leaf pieces were dispersed in a 200 mL Erlenmeyer flask containing 100 mL DI water. The dispersion was boiled using a magnetic stirrer at 500 rpm. The aqueous dispersion was then filtrated with Whatman filter paper to obtain the required leaf extract. Finally, the obtained leaf extract was stored at 4 °C for future use in the synthesis of the ZnO-Pd nanosheets.

### 3.3. Biosynthesis of ZnO-Pd Nanosheets

Zinc acetate dihydrate (2 M) and palladium chloride (0.0025 M) were placed in a 200 mL Erlenmeyer flask containing 50 mL DI water. The flask was kept on a magnetic stirrer at 850 rpm for 30 min. Thereafter, the magnetic stirrer temperature was set to 65 °C and 3 mL leaf extract was added dropwise after 10 min. After this, 50 mL (0.2 M) NaOH solution was added into it. The reaction was continued under the same conditions for 120 min. The obtained solution was then centrifuged at 3500 rpm for 15 min and the supernatant was removed. The obtained nanosheets were washed with DI water and redispersed into 20 mL DI water. The absorption peak was analyzed using UV vis spectroscopy (UH-5300, Hitachi, Ibaraki, Japan). Finally, the obtained solution was freeze-dried to obtain the dried form of ZnO-Pd nanosheets for various characterizations.

### 3.4. Characterization of Nanosheets

The absorption of the synthesized ZnO-Pd nanosheets was analyzed using a UV–vis spectroscopy (UH-5300, Hitachi, Ibaraki, Japan) device in the scanning range of 250–700 nm. The size and morphology of the synthesized ZnO-Pd nanosheets were analyzed using TEM (JEM-3010, JEOL, Japan). The freeze-dried nanosheets were analyzed using SEM (Zeiss, EVO 18, Akishima, Germany). The elemental composition of the ZnO-Pd nanosheets was evaluated using EDX. Furthermore, the XRD patterns were examined with an X-ray diffractometer (X’Pert PRO, PANanalytical, The Netherland) with CuK_α_ radiation (λ = 1.5417 Å).

### 3.5. Peroxidase Mimetic Activity

The synthesized ZnO-Pd nanosheets were used to analyze their peroxidase mimetic activity by performing catalytic oxidation of the peroxidase chromogenic substrate TMB. The solution of H_2_O_2_ (34.5%/d-1.135) was prepared by diluting it in DI water to bring the final working concentration to 20 mM. The TMB aqueous solution was prepared in dimethyl sulfoxide (DMSO). A typical reaction mixture contained 5 μL of 105 mM TMB, 50 μL of 400 mM H_2_O_2_, and in the absence or presence of 20 μL of 32 mM ZnO-Pd nanosheets. The remaining volume was maintained by adding acetate buffer (pH 4). The mixture solution was incubated at room temperature. The oxidized TMB produced a blue color and the reaction was measured by considering the absorption peak at 650 nm.

### 3.6. Determination of Catalytic Activity for TMB Oxidation

The amounts of Pd and Zn in the nanosheets were determined by inductively coupled plasma mass spectrometry (ICP-MS) in Korea Basic Science Institute (Daejeon, South Korea). The oxidase activity of ZnO-Pd was measured using 3,3′,5,5′-tetramethylbenzidine (TMB) as the substrate in the presence of excess H_2_O_2_. To determine the V_max_ value, ZnO-Pd (the final 10 μM of Pd) was added to sodium acetate buffer (0.1 M acetate buffer pH 5.0, 20% DMSO) containing H_2_O_2_ (final 80 mM) and TMB (final 1000, 500, 250, 125, 62.5, 31.25, 15.625 μM), at a final volume of 1 mL. The change in the light absorption at 650 nm, which results from TMB oxidation by H_2_O_2_, was measured for 3 min on UH5300 spectrophotometer (Hitachi, Tokyo, Japan) and the slope was converted to velocity (μM/min) using the extinction coefficient of oxidized TMB. The V_max_ value was determined by the Lineweaver–Burk plot.

## 4. Conclusions

In summary, we developed an eco-friendly, rapid, and cost-effective biological method for the synthesis of ZnO-Pd nanosheets. This is an unprecedented method developed for nanosheets. ZnO-Pd nanosheets were first found to have catalytic activities similar to those of the peroxidase enzyme. UV–vis absorption spectra of as-synthesized ZnO-Pd nanosheets exhibited two peaks, because one peak was removed by the formation of zero valent palladium. XRD patterns revealed 2θ values of 21.8°, 33.3°, 47.7°, and 56.2°, corresponding to the crystal planes of (100), (002), (112), and (201), respectively. The d-spacing of the palladium nanoparticles in the ZnO sheets was found to be 0.22 nm, which corresponded to the (111) plane. EDX analysis revealed that the biosynthesized ZnO-Pd nanosheets contained pure elemental Zn, O, and Pd. The ZnO-Pd nanosheets were observed to possess peroxidase mimetic activity, which oxidized the 3,3′,5,5′-tetramethylbenzidine substrate in the presence of H_2_O_2_. After oxidation, the colorless TMB acquired a dark blue color. Therefore, the present study demonstrates that the synthesized ZnO-Pd nanosheets exhibit peroxidase activity, which increases the reaction rate.

## Supplementary Materials

The following are available online. Figure S1: Green synthesis of ZnO nanosheets and ZnO-Pd nanosheets. (**a**) Ultraviolet–visible spectra of biosynthesized ZnO nanosheets. (**b**) White-yellow-colored dispersion of as-synthesized ZnO nanosheets. (**c**) Ultraviolet–visible spectra of biosynthesized ZnO-Pd nanosheets. (**d**) Light yellow-brown-colored dispersion of as-synthesized ZnO-Pd nanosheets. Figure S2: FTIR spectrum of biosynthesized ZnO-Pd nanosheets. Figure S3: Absorbance spectra of TMB and oxidized TMB after 20 min of incubation at room temperature. Table S1: The amounts of Pd and Zn in nanosheets were determined by ICP-MS.
