# Supplementary material for: Enzyme Mimetic Activity of ZnO-Pd Nanosheets Synthesized via a Green Route
Source: Molecules. 2020 Jun 2;25(11):2585. doi: 10.3390/molecules25112585 (PMC7321175; doi:10.3390/molecules25112585)
Supplement: Supplementary file 1 [file molecules-25-02585-s001.pdf]

molecules

MDPI

Article

Enzyme mimetic activity of ZnO-Pd nanosheets synthesized via green route

Ravi Mani Tripathi[1,2], Dohee Ahn[1], Yeong Mok Kim[1], Sang J. Chung[1]*

[1] School of Pharmacy, Sungkyunkwan University, 2066 Seoburo, Jangan-gu, Suwon, Gyeonggido 16419, Republic of Korea

[2] Amity Institute of Nanotechnology, Amity University Uttar Pradesh, Sector 125, Noida 201303, India

*Correspondence: sjchung@skku.edu; Tel.: +82-31-290-7703; Fax +82-31-292-8800

Received: date; Accepted: date; Published: date

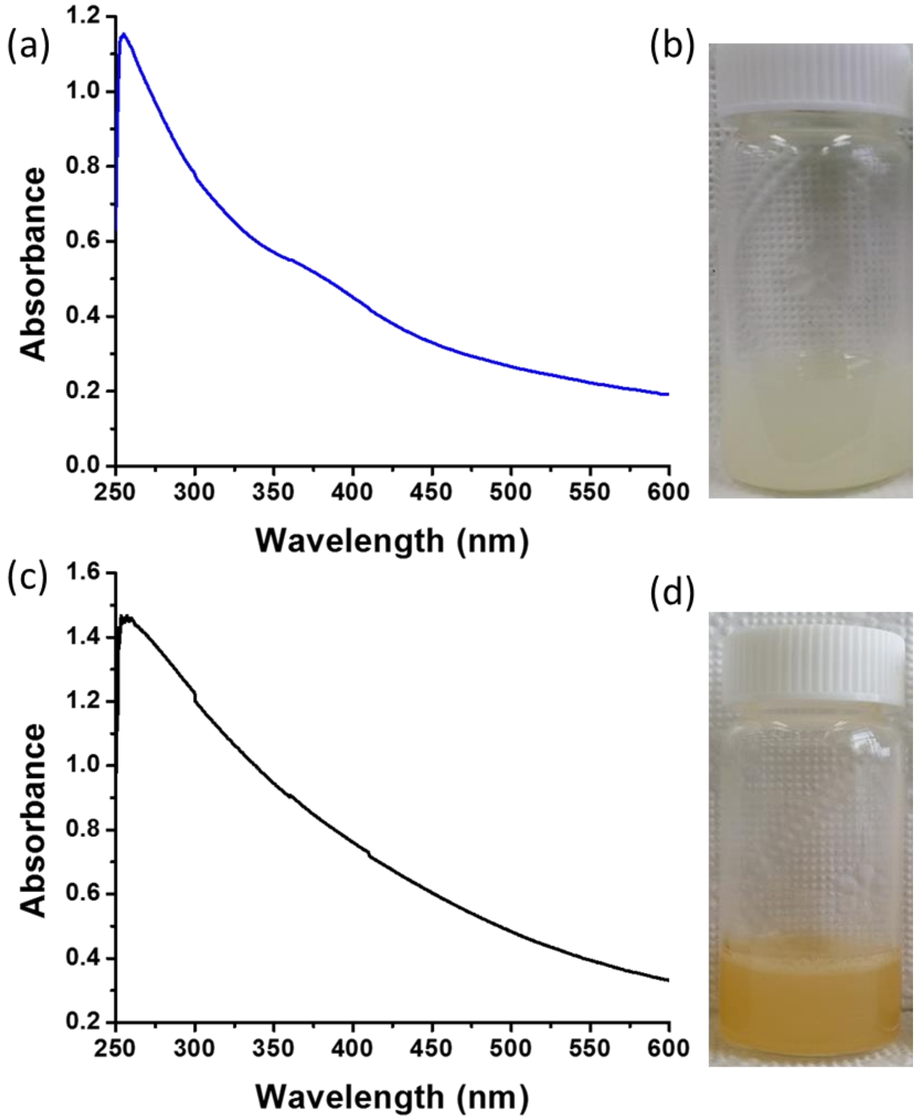

Figure S1. Green synthesis of ZnO nanosheets and ZnO-Pd nanosheets.

(a) Ultraviolet–visible spectra of biosynthesized ZnO nanosheets. **(b)** White-yellow-colored dispersion of as-synthesized ZnO nanosheets. **(c)** Ultraviolet–visible spectra of biosynthesized ZnO-Pd nanosheets. **(d)** Light yellow-brown-colored dispersion of as-synthesized ZnO-Pd nanosheets.

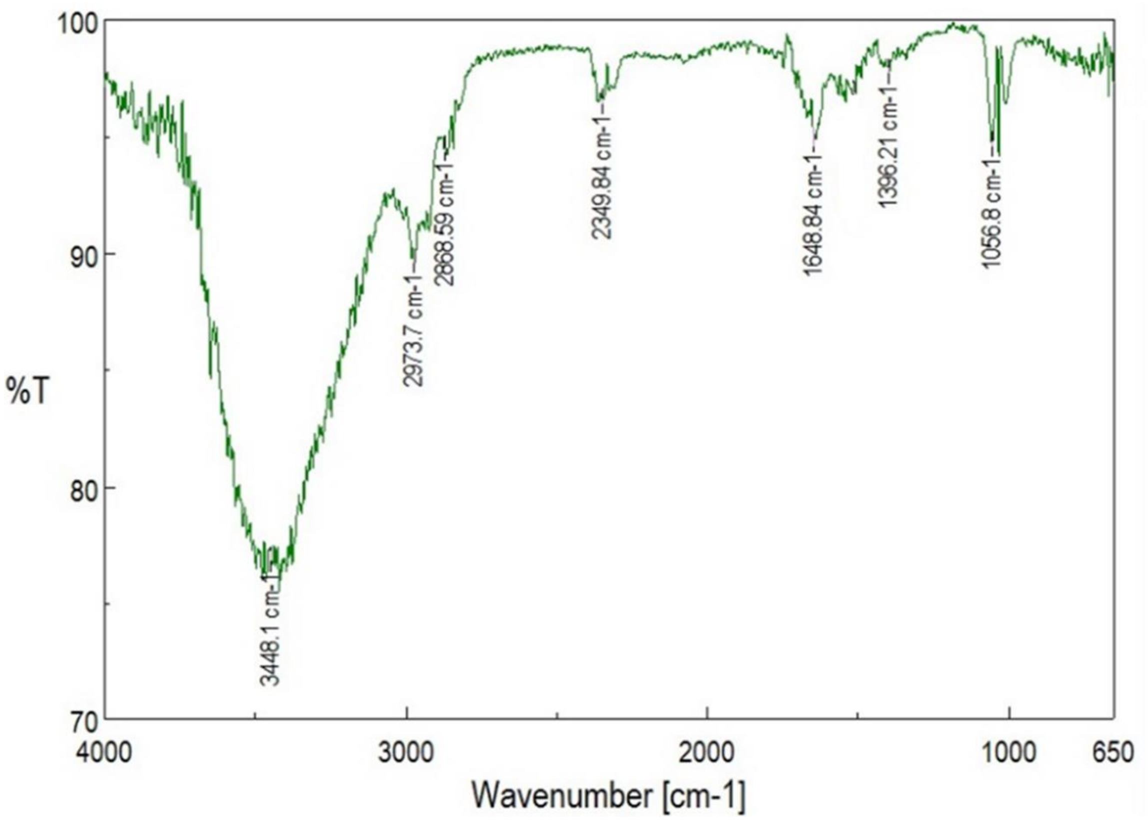

Figure S2. FTIR spectrum of biosynthesized ZnO-Pd nanosheets.

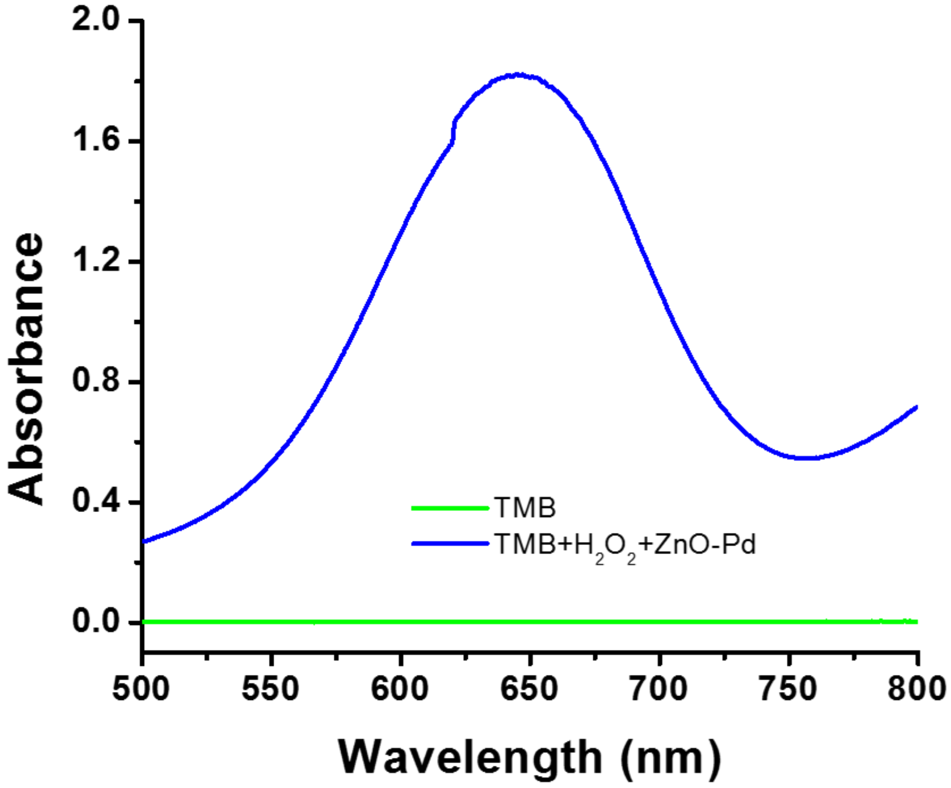

Figure S3. Absorbance spectra of TMB and oxidized TMB after 20 min of incubation at room temperature.

Table S1. The amount of Pd and Zn in nanosheets was determined by ICP-MS.

Element	Pd	Zn
% (w/w)	0.45	11.6
